# Fish-Ing for Enhancers in the Heart

**DOI:** 10.3390/ijms22083914

**Published:** 2021-04-10

**Authors:** Costantino Parisi, Shikha Vashisht, Cecilia Lanny Winata

**Affiliations:** 1 International Institute of Molecular and Cell Biology in Warsaw, 02-109 Warsaw, Poland; cparisi@iimcb.gov.pl (C.P.); svashisht@iimcb.gov.pl (S.V.); 2 Max Planck Institute for Heart and Lung Research, 61231 Bad Nauheim, Germany

**Keywords:** enhancers, heart development, heart regeneration, transcription factors, model organism, zebrafish

## Abstract

Precise control of gene expression is crucial to ensure proper development and biological functioning of an organism. Enhancers are non-coding DNA elements which play an essential role in regulating gene expression. They contain specific sequence motifs serving as binding sites for transcription factors which interact with the basal transcription machinery at their target genes. Heart development is regulated by intricate gene regulatory network ensuring precise spatiotemporal gene expression program. Mutations affecting enhancers have been shown to result in devastating forms of congenital heart defect. Therefore, identifying enhancers implicated in heart biology and understanding their mechanism is key to improve diagnosis and therapeutic options. Despite their crucial role, enhancers are poorly studied, mainly due to a lack of reliable way to identify them and determine their function. Nevertheless, recent technological advances have allowed rapid progress in enhancer discovery. Model organisms such as the zebrafish have contributed significant insights into the genetics of heart development through enabling functional analyses of genes and their regulatory elements in vivo. Here, we summarize the current state of knowledge on heart enhancers gained through studies in model organisms, discuss various approaches to discover and study their function, and finally suggest methods that could further advance research in this field.

## 1. Introduction

The cell specification and tissue remodeling that entails the formation of an organ are complex biological processes which are modulated by coordinated spatiotemporal execution of gene regulatory networks which dictates cell fates and organize specialized cell types into complex three-dimensional units of structure and function [1,2]. These networks are composed of diverse genes and their regulatory elements that evolve at different rates and can undergo various modifications. Non-coding DNA regulatory elements mediate the molecular networks of regulatory processes at the transcriptional, post-transcriptional and post-translational levels [3]. They include promoters [4], silencer [5], insulator [6], and *cis*- and *trans*-regulatory elements [7,8].

Enhancers are a major type of *cis*-regulatory element in the genome which increase the likelihood of transcription of one or more distally located genes [9]. The function of an enhancer was demonstrated for the first time in a non-coding region which contains two 72 base pair (bp) repeats of the simian virus 40 (SV40), which is able to drive efficient transcription of SV40 early genes [10,11,12,13]. Subsequently, it was found that enhancers were associated with genes that exhibit tissue-specific expression. The first cell type-specific enhancer was identified in mammalian B lymphocytes within the IgH locus [14,15,16]. Enormous progress has been made since the first discoveries about enhancer properties and their *modus operandi*. Enhancers serve as binding sites for transcription factors (TFs) which are necessary for the activation of target gene expression. Through chromatin looping, these regulatory factors are brought into direct physical contact with their target promoters [17,18] and thereby potentiate transcriptional initiation and elongation by interacting with the basal transcriptional machinery and the local chromatin remodeling of target genes [19,20]. More recently, it was found that active enhancer state is associated with the generation of bi-directional non-coding transcripts of the enhancer region known as enhancer RNAs (eRNAs) which are functionally required for its activity [20,21,22,23,24].

The heart is an essential organ which serves to circulate blood throughout the whole body. It is the first organ to form in the embryo. Heart development involves an extraordinary and precisely orchestrated series of molecular processes. The combination of complex morphogenetic events necessary for the formation of the heart results from an evolutionarily conserved gene regulatory network [25,26,27,28,29,30]. A core set of conserved cardiac TFs, such as *NK2*, *MEF2*, *GATA*, *Hand* and *Tbx*, is involved in the specification of cardiac cell fate, contractility, morphogenesis, segmentation, and growth of cardiac chambers [1,31]. This set, together with others TFs, govern and stabilize the developmental program of the heart [32,33,34]. Expansion and modifications of this ancestral cardiac network could give rise to hearts with higher structure and function complexity [35,36,37]. Homologs of the cardiac TFs were found in early multicellular organisms (~800 million years ago). These organisms possess a primitive coelom surrounded by cells which express *NKX2.5/tinman*, a TF necessary for the specification of cardiac fate in higher vertebrates [38]. The specialization of mesodermal cells around the coelom in the phylum Bilateria gave rise to the first primitive cardiac myocytes [39]. A further evolved heart structure is found in Protostomia (e.g., Drosophila) and Deuterostoma (e.g., Amphioxus), in the form of a peristaltic tubular heart arising from a monolayer of contracting mesoderm [38,40,41]. In the phylum Chordata and subsequently in the vertebrates, the primitive linear heart develops looping, unidirectional circulation, enclosed vasculature, and the conduction system. Fish and amphibians had further specializations with regional protein localization in cardiomyocytes, while, in reptiles, birds, and mammals, the heart is further sophisticated with the formation of septa and a four-chambered heart which has lost the ability to regenerate cardiomyocytes [38].

Cardiomyocytes (CMs) are the basic units of cardiac tissue which are specified from a pool of mesodermal progenitors located at the anterior portion of the embryonic lateral plate mesoderm [28,42]. Heart development initiates with the specification of cardiac cell fate with the expression of the homeobox TF *tinman* in invertebrates or *Nkx2.5* in vertebrates. This TF is considered the earliest molecular marker of heart progenitors and is known to interact with the zinc finger TF of the GATA family. In flies, *Tinman* directly activates *Mef2* gene, which encodes transcriptional elements that control myocyte differentiation [33]. The MADS-box protein MEF2 is the most ancient myogenic TF that, via specific combinations of *cis*-regulatory elements, regulates different muscle gene programs [1]. In zebrafish, the *tinman*-related gene orthologs *nkx2.5* and *nkx2.7* are responsible for the earliest step of cardiac genes initiation and regulate the expressions of *tbx5* and *tbx20* through the heart tube stage [43]. *Bmp* and *Nodal* signaling induce *nkx2.5* expression and cardiogenic differentiation by inducing *gata5* [44].

Following the specification of cardiac progenitors, the beating linear heart tube forms by the convergence of cardiac muscle cells along the ventral midline of the embryo. This linear cardiac structure is composed of extracellular matrix and myocardial and endocardial layers. The GATA family TFs play an essential role in forming the linear heart tube in both invertebrate and vertebrate species [45,46,47]. In vertebrate such as mice and zebrafish, the heart tube subsequently undergoes rightward looping which is essential for the alignment of the heart chambers. The rightward direction of cardiac looping is driven by asymmetric axial signals which include members of the transforming growth factor beta (*TGF-β*) family, Sonic hedgehog, and Nodal, which are expressed in the lateral mesoderm [44,48]. In mice, frogs, and in zebrafish, the TF *Pitx2* is involved in the mediation of left-right signals, which is expressed along the developing organs [48,49]. In addition, in zebrafish, the asymmetric heart looping is controlled by both Nodal-dependent and -independent mechanisms [50].

Despite the wealth of knowledge on TFs regulating heart development, very few enhancers to which they bind and exert their functions have been identified. As TFs bind to enhancers to exert their function, mutations in these regulatory elements can equally affect developmental outcome as mutations in coding regions. Studies in humans have revealed a number of such mutations associated with increased risk to various heart diseases (reviewed in [51,52,53,54]). The discovery of enhancers regulating multiple steps of heart development and regeneration has been greatly facilitated by the development of various approaches which allow their large-scale discovery. These include genome-wide profiling techniques such as chromatin immunoprecipitation followed by DNA sequencing (ChIP-seq) [20,55,56,57,58], computational predictions [59,60], and in vivo assay [61,62,63,64]. These approaches have been used on various model organisms such as the fruit fly *Drosophila melanogaster* [65], mouse *Mus musculus* [66,67], zebrafish *Danio rerio* [68,69], and the African clawed frog *Xenopus laevis* [70,71].

The zebrafish (*Danio rerio*) was established as a model organism for genetic studies by George Streisinger at the Oregon University in the late 1960s [72]. Several unique biological properties make zebrafish an attractive model for studying heart development. The embryos are transparent, allowing direct in vivo imaging of the developing heart. The zebrafish embryos are not fully dependent upon a functional cardiovascular system, which allows loss of function analysis up to a relatively late developmental stage compared to mammals. Despite having only two chambers, the zebrafish heart develops by means of a mechanism conserved with its mammalian counterpart [73]. The high number of offspring and low cost of maintenance of the zebrafish render it a good model for genomics studies, harboring the potential for rapid discovery of enhancers and other genetic regulatory elements involved in heart development [44,69,74]. The zebrafish entered the genomic field relatively late compared to mammalian and other model organisms as evidenced by the lack of systematic efforts such as ENCODE [75] and modENCODE [65,76] to study its regulatory genetic elements. Nevertheless, such efforts are underway (DANIO-code) and more and more high-throughput techniques are employed to explore its genome [77]. Here, we summarize the advances in cardiac enhancers discovery and how it has contributed to elucidating multiple aspects of heart development and function. We particularly focus on the zebrafish as a convenient model for in vivo study of enhancer functionality. We also review emerging techniques which could be applied for cardiac enhancer discovery and functional study in the future.

## 2. The Quest for Enhancers Involved in Heart Development and Function

### 2.1. Targeted Analysis of Gene Promoter Regions Pinpoints Regulatory Elements Driving Tissue-Specific Expression

Functional characterization of enhancers was traditionally performed by deletion mapping, in which putative regulatory regions upstream of transcription start sites (TSS) of candidate genes are tested by means of a reporter assay to determine its ability to drive specific expression pattern (Table 1). In the zebrafish, these reporter assays can be done in vivo, where a transgenic reporter construct carrying a green fluorescent protein (GFP) reporter can be directly injected into the embryo and its resulting expression observed live. In one of the earliest applications of the deletion mapping strategy in zebrafish, Meng and colleagues [78] characterized the promoter region of the zebrafish *gata1* gene which encodes a TF that plays an important role in hematopoietic development. Using transgene constructs containing deleted and point mutated regions of the *gata1* promoter previously identified in zebrafish [79], they identified distinct *cis*-acting elements that regulate *gata1* transcription in various tissues. Systematic deletion on the 5.6 kb genomic fragment upstream of the zebrafish *gata1* translation start codon, identified a CACCC box, located between −146 and −142 bp upstream of the translation start codon, which were critical for the initiation of *gata1* expression. Furthermore, they showed that the hematopoietic expression of *gata1* was maintained by double GATA motif in the distal region between −4635 and −4627 bp. In addition, a 49 bp element located 218 bp upstream of the CACCC element and a CCAAT box at −4643 bp adjacent to the double GATA motif enhanced the erythroid-specific activity of *gata1* promoter. Interestingly, a region located between −1776 and −468 was found to repress the *gata1* expression in the notochord (a nonhematopoietic tissue), making it one of the earliest discoveries of a repressive hematopoietic regulatory element in the zebrafish. In a similar approach, Muller and colleagues [80] investigated the *cis*-regulatory elements directing sonic hedgehog (*shh*) expression in the zebrafish embryo. They focused on the zebrafish *shh* region, employing an enhancer screening strategy based on co-injection of putative enhancer sequences with a reporter construct [81]. They identified three regulatory elements in introns 1 (ar-A and ar-B) and 2 (ar-C) that mediated floor plate and notochord expression. Deletion fine mapping strategy on ar-C delineated three sub regions of 40 bp essential for its activity. A T-box TF binding site was found in one of these subregions, but none of the three regions contained binding sites of *Foxa2,* which was previously shown to regulate of *shh* expression [82], suggesting that other regulatory mechanisms were involved in *shh* expression. Importantly, these enhancers were able to drive similar expression pattern in zebrafish as well as in mouse embryos, showing that the mechanism controlling shh expression in the midline were evolutionarily conserved. Both these studies therefore showed for the first time that the transient enhancer expression assay using zebrafish embryos could be exploited to identify novel regulatory elements in gene fragments.

The enhancer deletion mapping approach was applied to study the regulatory mechanism of *gata4* [83], a TF which plays an essential role in specification of cardiomyocytes and formation of the linear heart tube [45,46,47]. A 14.8 kb fragment upstream of the *gata4* transcription initiation site was found to drive GFP expression in both chambers and the valves of the zebrafish heart. Truncation of 7 kb of the distal sequences eliminated expression in the atrium and the atrioventricular valve, while expression was retained in the ventricle and bulboventricular valves. Within this 7 kb distal regulatory region, a 1300 bp region with a cluster of consensus binding sites for T-box TFs was delineated. Mutation of these binding sites significantly reduced reporter gene expression in the heart, providing the first evidence that T-box factors function by directly regulating *gata4* expression. This study established that *gata4* regulatory elements control gene expression differentially along the rostro-caudal axis and that T-box binding elements in the *gata4* promoter contribute to heart-specific expression.

### 2.2. Large-Scale Enhancer Discovery by Enhancer Trapping Generates Live Markers for Developmental Studies

**Figure 1:**
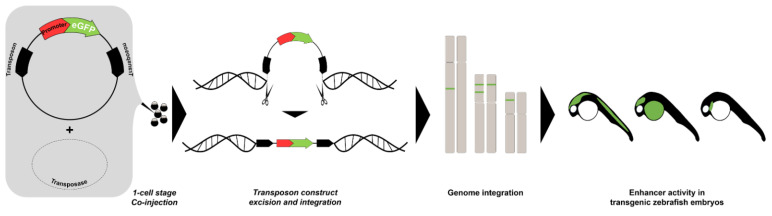
Enhancer trapping method. The synthetic transposase mRNA, the transposon donor plasmid containing transposable elements with a minimal promoter, and the gene encoding green fluorescent protein (GFP) are co-injected into fertilized zebrafish eggs. The construct is excised from the donor plasmid and integrated into the endogenous genome. The activity of the “trapped” enhancer can be visualized in the injected embryo when the inserted transgene is expressed under control of nearby enhancers.

To perform large-scale discovery of enhancers, several methods were developed in model organisms, particularly the zebrafish (Table 1). One of these methods, known as enhancer trapping, offers ease of genetic manipulation by transgenesis (Figure 1). In 2000, Kawakami and colleagues developed a gene trap method in zebrafish using a modified *Tol2* transposable element isolated from medaka fish (*Oryzias latipes*), which encodes a gene for a fully functional transposase capable of catalyzing transposition in the zebrafish germ lineage [84]. The *Tol2* element could be inserted in the zebrafish genome and transmitted to the next generation with a low transgenic frequency which prevented to generate hundreds or thousands of transposon insertions. In 2004, they further optimized the *Tol2* system, incorporating the *Xenopus EF1α* enhancer/promoter, the rabbit *β-globin* intron, the enhanced green fluorescent protein (*EGFP)* reporter gene, and the SV40 polyA signal, which results in EGFP expression that could be observed consistently up to the *F4* generation [85]. This established a highly efficient transgenesis method with more than 50% of frequency compared to other methods using naked plasmid DNA or other transposon systems. Applying this technique, Kawakami and colleagues established a collection of transgenic zebrafish lines with a variety of EGFP expression patterns, including ubiquitous, and spatiotemporally restricted patterns. Unique reporter expression patterns were detected in the heart, forebrain, notochord, floor plate, neural crest, and other tissues, establishing a novel transposon-mediated gene trap approach in zebrafish and facilitating studies of vertebrate development and organogenesis. In a parallel effort, Parinov and colleagues also reported the application of an enhancer trap approach in zebrafish using the Tol2 transposon system [86]. They used an enhancer trap construct that carried the *EGFP* gene as reporter in live zebrafish embryos controlled by a partial promoter of the epithelial *keratin4* (*krt4*) gene. In their initial screen, 37 founders (*F0*) transmitting the actively expressed *EGFP* gene to their offspring (*F1*) were identified. These founders were raised, outcrossed with wild-type, and analyzed up to the *F2* generation. In total, they established 28 enhancer trap (ET) lines that exhibited distinct EGFP expression patterns apart from the basal expression from the modified *krt4* promoter. The EGFP fluorescence was detected in a variety of tissues and organs, including the central nervous system (CNS), neural crest and its derivatives, notochord, heart, muscles, digestive organs, and kidney. This study, together with that of Kawakami and colleagues, demonstrated that the enhancer trap construct could produce high trapping frequency and specificity.

Subsequently, Poon and colleagues [61] further screened the collection of zebrafish enhancer trap lines [86,87,88] and found 18 cardiac enhancer trap (CET) lines with EGFP expression in various part of the embryonic heart. They characterized the EGFP expression pattern in the embryonic heart in vivo using fast scanning confocal microscopy coupled with image reconstruction, producing three-dimensional movies in time. The transgenic lines exhibited EGFP expression in distinct cell layers of the heart, including the endocardium, myocardium, and epicardium. Subsequently, the genomic locations of the transposon insertions were identified by thermal asymmetric interlaced polymerase chain reaction (TAIL-PCR). This screen therefore established a collection of CET lines which could be utilized as a starting point for discovery of cardiac enhancers through further analysis. Furthermore, the cardiac EGFP expression is useful for in vivo studies of heart development.

Balciunas and colleagues [97] modulated the salmonid-originating *Sleeping Beauty* (SB) transposon-based transgenesis cassette [98] to establish an enhancer trapping in zebrafish. It belongs to the Tc1/mariner superfamily of transposons consisting of two components: the transposase enzyme and a transposon vector containing the terminal-inverted repeat/direct repeat (IR/DR) sequences which moves by a cut-and-paste mechanism. Optimization of this system allowed them to establish nine transgenic lines (ET1–ET9) with different tissue-specific patterns including various tissues of the nervous system, otic vesicle, and the heart in one of the lines (ET7). Detailed analysis on lines ET2 and ET7 revealed that the ET2 line harbors a transposon insertion in the gene encoding poly(ADP-ribose) glycohydrolase (PARG) expressed in caudal primary motoneurons. The GFP expression in the ET2 line recapitulated that of the endogenous gene (PARG), indicating that transgene expression was under control of an endogenous enhancer. The ET7 line had closely resembled part of the *dual specificity phosphatase 6* (*dusp6)* expression domain, which is expressed in the midbrain-hindbrain boundary, forebrain, tailbud, branchial arches, developing ear, pectoral fin buds, and other tissues [99], suggesting that the enhancer trap transposon in that line was under control of a subset of *dusp6* enhancer elements.

Despite its potential to discover a large number of enhancers, a major drawback of the enhancer trap approach is that the enhancers driving the specific expression are not always easy to identify. Only in some cases, analyses of expression by whole-mount RNA in situ hybridization demonstrated that EGFP expression patterns in enhancer trap lines reflected the tissue-specific expression of nearby genes. Nevertheless, these lines provide a valuable starting point for assays such as chromatin conformation capture, which would allow the identification of the enhancer responsible for driving the reporter expression. In addition, the enhancer trapping screens has generated a wealth of resources in the form of live transgenic markers for various tissues, which is useful for developmental studies.

### 2.3. Comparative Genomics Identify Highly Conserved Developmental Enhancers Regulating Heart Development

It is well established that enhancers regulating critical developmental processes are under strict evolutionary constraint due to their critical function to ensure an organism’s viability (Table 2) [100,101,102]. Comparison of multiple genomes by homology sequence analysis therefore provides a promising method to determine and characterize novel developmental enhancers, including those which play a role in heart development. Several studies in the past have implemented evolutionary conservation as a successful criterion to identify tissue-specific developmental enhancers [103,104,105,106,107,108]. In one of the earliest examples, Aparicio and colleagues [103] applied the principle of sequence conservation between highly divergent vertebrates (~430 million years), in which alignment between *Fugu rubripes* (Japanese pufferfish) and mouse identified the existence of three conserved blocks within intronic and 3′-non-coding regions of the *Hoxb-4* gene locus which could drive gene expression in mesoderm, peripheral, and central nervous systems in transgenic mice. Pennacchio and colleagues [109] implied genome-based comparative frameworks, focusing on finding large sets of enhancers genome-wide in forebrain tissue of mouse embryos at e11.5 developmental stage. Based on whole-genome pairwise sequence comparison between highly divergent (human and *Fugu*) and fairly divergent (human and mouse) species, they identified conserved and ultra-conserved non-coding elements containing the binding sites of various TFs which were corroborated using in vivo transgenic enhancer assay in mouse.

Applying the same principles, Wang and colleagues [62], using the zebrafish genome as basal genome, identified evolutionarily conserved regions that had a minimum similarity of 70% between the zebrafish (zv9) and human (hg19) genomes with a minimum length of 100 bp. They focused their analysis on a region of 150 kb upstream and downstream of *notch1b* TSS—the zebrafish ortholog of *NOTCH1*. The Notch pathway plays important roles in cardiac development, heart differentiation, and proliferation of immature cardiomyocytes [110]. Initially, they found a 127 bp conserved sequence located approximately 85 kb downstream of the *notch1b* TSS, which drove GFP reporter expression in the embryonic heart. Subsequently, they employed a deletion strategy to identify the core region supported by TF prediction and core TF distribution analyses. A 42 bp short fragment overlapping the 35th exon of the gene *si:dkey-21e5.1* was found to be sufficient for driving the heart expression. Overexpression in cells further showed that a mutation of the *NKX2.5* binding site within this region significantly decreased reporter gene expression, underlying its critical role on the enhancer activity in the heart. This study revealed a short heart-specific enhancer of *notch1b* which may play a role in zebrafish heart tube formation and heart maturation. This enhancer, regulated by NKX2.5, is located in a coding exon of a nearby gene, showing a new type of enhancer in heart gene regulation already found in other organism or tissues [111].

Zhang and colleagues [64] followed a similar approach, comparing zebrafish and human genome. They identified enhancers located within 100 kb regions upstream and downstream of the *tnni1b* TSS. They focused on a region of 219 kb genomic range, covering the *tnni1b* gene, a zebrafish ortholog of *TNNI1*. This gene, which is important for the formation of the atrioventricular canal in zebrafish hearts [112], together with *troponin T* (*tnnt*) and *troponin C* (*tnnc*), are subunits of a complex that regulates cardiac and skeletal muscle contraction [113]. A 183 bp conserved sequence located approximately 84 kb upstream of the *tnni1b* TSS was found to drive GFP expression in the embryonic heart. To identify the core region responsible for enhancer activity, the authors further implemented TF prediction and core TF distribution analyses [114], which aligns the sequence of TF binding sites found on two orthologous DNA sequences from two species. An 87 bp short fragment was identified and found to be sufficient for the enhancer activity in the heart, showing specific GFP expression near the atrioventricular junction of the heart. Analysis of TF binding sites within this region further revealed known cardiac TF candidates which may regulate this enhancer, including Nkx2.5 and Jun. As expected, an increase of enhancer activity as measured by in vitro luciferase assay was observed when NKX2.5 or JUN was overexpressed and a decrease when their putative binding sites were mutated. These results demonstrate that *tnni1b* expression is directly regulated by Nkx2.5 and Jun by means of the identified enhancer 84 kb upstream of the TSS, which may be implicated in atrioventricular valve development.

The two abovementioned studies underlined the role of conserved enhancers in the heart, showing how evolutionary conservation could be used as a reliable indicator to identify critical enhancers regulating developmental function. In several other studies, comparative analyses were performed between organisms other than zebrafish to detect evolutionarily conserved regions (ECRs) in the heart. The zebrafish system allows live enhancer assay, and conservation of enhancer function between zebrafish and mammals has been demonstrated [58,62,64,101,115]. Due to these features, many candidate enhancers identified in the mammalian system were validated in the zebrafish system [57,63,108,116,117]. For example, Woolfe and colleagues [108] identified 1373 highly conserved non-coding elements (CNEs) conserved between Human and *Fugu* genome, including 25 vertebrate-specific highly conserved non-coding sequences that were located around four unrelated developmental regulators, SOX21, PAX6, HLXB9, and SHH. The enhancer activity of these CNEs were subsequently validated with GFP reporter assay in zebrafish embryos, revealing that 23 of them showed significant enhancer activity in one or more tissues, including a conserved region which directed expression in the blood and pericardium. In this way, the combination of a comparative genomics approach together with functional assay in zebrafish identified vertebrate-specific highly conserved enhancers related to developmental regulators which drove GFP expression patterns in nervous system, sensory organs, notochord, muscle, blood islands, heart, and skin. The correlation between the expression of endogenous gene and that induced by the enhancers suggests the role of these enhancers as part of gene regulatory network that defines vertebrate development.

### 2.4. Genome-Wide Enhancers Discovery Generates Valuable Resource on Gene Regulation in Heart Development and Function

The rapid advancement in genomics technology has greatly facilitated the discovery of enhancers (Table 1). The emergence of next-generation sequencing (NGS) technology more than 10 years ago has facilitated the expansion of genomics, increasing its output capacity and applicability in various biological disciplines [118,119]. Using chromatin immunoprecipitation coupled with NGS (ChIP-seq [120]), it is possible to profile epigenetic states which could reliably distinguish various genomic features, including active or inactive/poised enhancers. For instance, tri-methylation and mono-methylation of K4 at H3 (H3K4me3 and H3K4me1), indicates the presence of promoters and enhancers, respectively [90,91,92,93]. The hallmarks of functionally active enhancers are established with the presence of mono-methylated histone H3 modification at lysine 4 (H3K4me1) together with acetylation of lysine 27 residue of H3 histone protein (H3K27ac) and enrichment of histone acetyltransferase P300. Histone modification H3K4me1 alone marks the enhancer regions, while H3K27ac demarcates the active enhancers from inactive ones. Inactive enhancers are marked with the presence of tri-methylated histone H3 modification at lysine 4 (H3K4me3, which indicates the existence of promoters). The ability to identify enhancers genome-wide using epigenomic profiling has driven large-scale efforts to annotate human non-coding genomic regions. A high-quality comprehensive analysis of the human genome was provided by the ENCODE project, defining the functionality of 3 billion nucleotide bases [75]. In the pilot phase (2003–2007), the consortium analyzed ~1% of the human genome, providing in-depth insights into the functional regulatory elements, including *cis*-regulatory DNA regions, distal or long-range interacting enhancers, and TF binding regions [121]. Following the successful completion of the pilot phase, 500 ChIP-seq datasets within 70 different cell-types were generated to capture the diversity across human chromatin landscapes [122,123].

The field of heart biology has also benefited from this consortium effort. Within the framework of the ENCODE project, Dickel and colleagues [60] developed an extensive repository of more than 80,000 putative cardiac enhancers from mouse and human pre- and postnatal heart tissues by performing an integrative analyses of more than 35 published and unpublished ChIP-Seq datasets (deposited in GEO [124], ENCODE [75,122,123,125], and NIH Roadmap Epigenomics Consortium [126]), focused on enhancer-specific epigenetic marks (H3K27ac and P300/CBP (CREB-binding protein)). To achieve high confidence in putative enhancer identification, the authors further combined data from other methods known for enhancer prediction, including DNase hypersensitive sites (DHS) and TF binding sites for human and mouse heart samples. Most peaks obtained from DHS and TFBS data analyses overlapped with ChIP-seq enhancer genomic coordinates. Based on the ChIP-seq peak strength, a scoring schema was applied for accurate enhancer discovery. In total, 82,119 putative enhancers were identified; among them, 3677 were long enhancers ranging 5–9 kb in size. Two cardiac enhancer knockouts were generated in mouse model system to analyze their impact on nearby key cardiac disease-causing genes: *Myh7* and *Myl2*. The loss of either of these enhancers in homozygous null mice resulted in a decreased RNA expression levels of both genes by 75–85%. Successively, their deletions also caused cardiac deformities, including misalignment of cardiac myocytes (referred as myocardial disarray) and karyomegaly. These results imply the importance of identified enhancers in the normal development of heart and its functionality.

The earliest applications of NGS for the identification of genome-wide heart enhancers utilized ChIP-seq to isolate and sequence genomic regions bound by the P300 enhancer-associated protein in mouse and human heart tissue samples. To identify heart enhancers genome wide, Blow and colleagues [55] used ChIP-Seq targeting the enhancer-associated protein P300 in mouse embryonic heart tissue at Embryonic Day 11.5 (e11.5), when the heart undergoes chamber formation [127]. They identified 3597 regions bound by P300, which were considered candidate cardiac enhancers. They compared this result with P300-bound regions from other tissues, including forebrain (2759 enriched regions), midbrain (3839 enriched regions), and limb (2786 enriched regions), observing that the 84% of P300 peaks were exclusively present in the heart. Interestingly, evolutionary conservation analyses revealed that the predicted heart enhancers were more divergent compared to that of forebrain. Subsequently, 97 out of 130 candidate heart enhancers were validated in the transgenic mouse enhancer assay, where they were found to drive reproducible tissue-specific expression in e11.5 embryos. Moreover, the vast majority (81/97, 84%) were also active in the developing heart. Notably, enhancers identified in this study exhibited highly restricted expression pattern such as the interventricular septum, with 51/81 (63%) driving reporter gene expression exclusively in the developing heart. Collectively, based on comparison of enriched regions between the heart, forebrain, midbrain, and limb, the results of this study suggest that a large population of heart enhancers were poorly conserved and show that embryonic enhancers can vary in terms of evolutionary conservation depending on tissue type.

Using a similar approach targeting the P300, May and colleagues [56] performed chromatin immunoprecipitation with a pan-specific antibody that recognizes both P300 and the closely related CBP co-activator protein to identify enhancers directly from fetal and adult human heart tissue. By massively parallel sequencing and enrichment analysis, 5047 fetal and 2233 adult putative cardiac enhancer regions located at least 2.5 kb from the nearest TSS up to 200 kb away from promoters were identified. The 48% of adult heart enhancer candidates coincided with candidate enhancers derived from fetal human heart. Interestingly, 81 human fetal heart candidate enhancers were located within 50 kb of a set of genes used in genetic diagnosis of heart diseases. These genes were associated with a variety of cardiac diseases, including conduction disorders, cardiomyopathies, and congenital heart disease. Utilizing a transgenic mouse enhancer assay, 43 out of 65 candidate enhancers were validated, driving reproducible expression in the heart or vasculature, either exclusively (28, 43%) or as a part of reproducible compound patterns that included the heart (15, 23%). The genome-wide discovery of distant-acting enhancers in mammalian heart, as illustrated by the two abovementioned studies, is a valuable resource for downstream studies of regulatory elements in developmental and pathological conditions of heart.

In an attempt to dissect the molecular mechanism of cardiomyocyte specification, Wamstad and colleagues [20] defined the dynamic epigenetic and transcriptional landscapes in four stages of cardiomyocyte differentiation from mouse embryonic stem cells (ESCs) to cardiomyocytes (CM), including developmental intermediates as mesoderm (MES) and cardiac precursors (CP). They analyzed global expression patterns of polyadenylated transcripts and microRNAs (miRNAs) in the four cell types and identified 13,500 genes and over 600 miRNAs. In addition, long non-coding RNAs (lncRNAs) were found in stage specific expression, which were significantly correlated in expression with their neighboring genes. Interestingly, several correlated lncRNA–gene pairs involved known cardiac genes such as *Gata6*, *Hand2,* and *Myocd*. They then employed ChIP-seq to map histone modifications, including H3K4me1 and H3K27ac that demarcate enhancer elements in a wide range of cell types. They identified 81,497 putative distal enhancer regions during cardiac differentiation which they classified as active (H3K27ac+ and H3K4me1+/−) or poised (H3K4me1+ only) at each stage of differentiation. One of their interesting observations is that transitions between poised and active enhancer states occurred rapidly between stages of cardiomyocyte differentiation, in which the subpopulation of active enhancers that transited through a poised state was largest during the MES to CP and CP to CM transitions and lowest between unrelated cell types. They observed that motifs for TFs that drove cardiac development were enriched in active enhancers; these include OCT4, LRH1, GATA, MEF, MEIS1, and SRF. Interestingly, MEIS and GATA motifs were often enriched in the same enhancers and these were associated with genes important for cardiac development, and conduction system function. Five such enhancers were tested using luciferase reporter activation. They were synergistically activated by the combination of MEIS1A and GATA4, showing that GATA4 and MEIS1A could function together to activate certain cardiac enhancers. Thus, the analysis of chromatin state transition during cardiomyocyte differentiation in mouse embryonic stem cells revealed the correlation between enhancer activity and specific developmental programs during the process of cardiomyocyte differentiation.

### 2.5. Dynamics of Chromatin Landscape during Cardiogenesis Reveals Enhancers Implicated in Heart Development

Active enhancers are characterized by their open chromatin conformation which allows access to *trans*-acting regulatory factors. This feature has been commonly exploited to discover enhancers by profiling genome-wide chromatin accessibility, from which information on TFs binding within these locations along with nucleosome occupancy surrounding them could be derived. An early example of such technique utilizes deoxyribonuclease I (DNase I) digestion to cleave nucleosome-depleted, open, and accessible chromatin regions. These DHSs can be localized either by microarray-based methods [128,129] or high-throughput sequencing techniques [89] to identify functional *cis*-regulatory elements, including enhancers, in biological systems. Later, in 2013, Buenrostro and colleagues [130] developed an alternative method to profile open chromatin regions at genome-wide scale. The method is called assay for transposase-accessible chromatin using sequencing (ATAC-seq), which runs on the principle of targeting and fragmenting the accessible chromatin locations by employing Tn5 transposase and simultaneous adapter insertion for high-throughput sequencing. It requires significantly lower input cell numbers as compared to DNase-seq, and the short fragments generated in ATAC-seq precisely indicate transcriptionally active regions that are enriched with TF binding motifs/sites, bound by specific TFs. By analyzing ATAC-seq data, robust TFBS footprints can be generated to further identify putative distal *cis*-regulatory elements including enhancers.

Recently, we capitalized on the power of genomics analyses and the zebrafish model system to capture the dynamics of regulatory landscape and gene regulatory network throughout the progression of zebrafish heart morphogenesis in vivo [58]. We combined transcriptome profiling (RNA-seq) with an assay for chromatin accessibility (ATAC-seq) on isolated CMs from wild-type zebrafish heart at key stages of heart morphogenesis corresponding to linear heart tube formation, chamber formation/differentiation, and heart maturation. Among 50 genes with the highest average expression across all developmental stages, several were associated with human cardiac diseases including cardiomyopathy (*ttn.1*, *mybpc3*, *ttn.2*, *acta1b*, and *actn2b*), atrial septal defects (*actc1a* and *myh6*), and Laing distal myopathy (*vmhc*). Gene regulatory networks assembled based on RNA-seq expression profiles identified five regulatory modules enriched in functional terms involved in embryonic heart tube development, cardioblast differentiation, heart valve development, and heart formation. These contained genes encoding TFs previously implicated in heart development, such as *gata1*, *tbx5a*, *sox10*, *hand2*, *smad7*, *gata5*, *nkx2*.5, and *tbx20*. ATAC-seq analysis revealed a large number of genome-wide nucleosome free regions (NFRs) common to all stages (16,055). The most stage-specific NFRs were found at 24 hpf (22,656) and the highest fraction was localized within promoter, intergenic and intronic regions, revealing a strong link between chromatin accessibility of promoter regions and gene expression levels. Gene-distal-located NFRs were identified as potential distal transcriptional regulatory elements and were compared with the database of highly conserved non-coding elements between zebrafish and human. In total, 22 regions were found conserved between zebrafish and human genomic sequences. Among them, three were downregulated in *tbx5a* and *hand2* mutants, whereas 19 of them showed significantly increased accessibility in *hand2* and *gata5* mutants. These represent evolutionarily conserved enhancers candidates which potentially regulate critical steps of heart morphogenesis. Finally, zebrafish mutants deficient in cardiac-related TFs (Gata5, Hand2, and Tbx5a) showed dysregulation of numerous genes and differential accessibility of the NFRs, providing a strong validation of the cardiac regulatory networks controlling specific processes of heart development.

Enhancers represent sites where TFs bind to regulate the expression of downstream target genes. Therefore, combinations of TF binding patterns analysis with open chromatin regions can be employed to delineate enhancers and provide valuable information on their possible function. Van den Boogaard and colleagues [63] identified and characterized a transcribed distal enhancer involved in regulating the expression of cardiac ether-a-go-go-related gene (*hERG* or *KCNH2*). This gene encodes the voltage-gated potassium channel involved in the repolarization phase of the action potential in human cardiomyocytes [131]. Common variants in non-coding genomic regions close to *KCNH2* are known to be associated with cardiac arrhythmia [132,133]. The authors first predicted CREs candidates in both human and mouse *Kcnh2* locus by integrating available (ChIP-seq) datasets of cardiac TFs and dataset of proteins associated with active regulatory sequences and active transcription. Eleven candidate CREs were tested by in vitro luciferase assay in HL-1 cell line, a mouse atrial cardiomyocyte-like cell line, and in zebrafish in vivo enhancer assay. One of the CREs (CRE11) located ~85 kb downstream of the TSS of *Kcnh2* was identified as a promising candidate to regulate *Kcnh2* expression, displaying the strongest regulatory potential in vitro as well as in vivo. This region was previously found to be directly bound by multiple TFs including TBX20, which control the expression of *KCNH2* in human cardiomyocytes [134,135]. High-resolution chromosome conformation capture sequencing (4C-seq) further confirmed that CRE11 is in close spatial proximity to the promoters of both *Kcnh2* isoforms and of *Nos3*. As enhancer activity could be accompanied by its bidirectional transcription [94,136], transcriptional activity at CRE11 location were investigated. Using several strand-specific oligonucleotide sets on both sides of CRE11, they found that CRE11 eRNA were polyadenylated and transcribed in a bidirectional manner. In addition, antisense oligonucleotides-mediated knockdown of CRE11 eRNA in HL-1 cells resulted in downregulation of *Kcnh2b* as well as neighboring genes *Nos3* and *Abcb8*. Further analysis using CRISPR/Cas9-Mediated deletion of CRE11 in the mouse genome resulted in reduction of *Kcnh2a* and *Kcnh2b* expression in the ventricles. Combining this evidence, the study showed direct regulation of *KCNH2* expression by the CRE11 enhancer element which involved eRNAs expression. These results pave the way for future research on the mechanism of action of enhancers and their eRNAs.

Using chromatin accessibility information, Galang and colleagues [96] identified novel enhancers involved in the sinoatrial node (SAN) pacemaker gene regulation, development, and function. They performed ATAC-seq to compare regions of accessible chromatin in sorted cardiac pacemaker cells (PCs) and right atrial cardiomyocytes (RACMs). Interestingly, 108 out of the top 500 differentially accessible ATAC-seq peaks were associated with genes that were differentially expressed in PCs versus RACMs. Moreover, differentially accessible ATAC-seq peaks were enriched for binding motifs of known cardiac TFs, including Isl1, which is known for its role in PC development, and Meis1 involved in cardiac development. In addition, the PC-enriched subset of differentially accessible ATAC-seq peaks showed robust enrichment for Mef2c, Tbx5, and Gata4 motifs. Five candidate enhancers identified from the intersection of ATAC-seq peaks with embryonic mouse heart H3K27Ac ChIP-seq datasets from ENCODE [123] exhibited cardiac activity in consistent patterns in mouse embryos, of which three had activity in the SAN primordium and two were specific for the venous inflow. To search for additional enhancers, the authors surveyed the loci encoding known pacemaker TFs Shox2, Tbx3, and Isl1 for differentially accessible ATAC-seq peaks within previously annotated TADs (topologically associated domains). A single previously uncharacterized peak was identified, located ~20 kb downstream of *Isl1* (termed *Isl1* locus SAN enhancer, ISE). The ISE drove restricted reporter activity in the SAN at different developmental stages in stable transgenic mice, showing signals in cardiac inflow at embryonic stage, having a remarkable degree of specificity for the SAN throughout development and maturation. Deletion of ~2.7 kb ISE in mice using CRISPR/Cas9 led to a reduction of *Isl1* expression in SAN and abnormal SAN development, a reduction in PC proliferation, sinus arrhythmias, and slower heart rate. The mouse ISE exhibited > 70% conservation compared to humans and opossum, suggesting that the regulatory network upstream of this enhancer may also be deeply conserved. Remarkably, the mouse ISE was also conserved in function in the zebrafish as it was able to drive robust reporter expression in the junction of the sinus venosus and atrium, consistent with deep evolutionary conservation of the regulatory network controlling enhancer activity. A set of human genomic regions syntenic to the murine ATAC-seq peaks in RACMs and PCs was identified, showing multiple single nucleotide polymorphisms (SNPs) associated with resting heart rate in close proximity to ISE, providing evidence for their functional role in human SAN. In addition to the ISE, 19 other fragments containing binding sites of cardiac TFs Gata4, Tbx5, and Tead were also demonstrated to drive reporter expression in the entire zebrafish heart and sinus venosus without restriction of enhancer activity to the SAN region, suggesting a model in which the Gata4, Tbx5, and Tead may be sufficient for enhancer activation but are insufficient to confer enhancer specificity to the SAN. Altogether, the epigenetic profile of cardiac SAN PCs was defined and a novel set of SAN enhancers was discovered and validated. These include a deeply conserved PC-specific enhancer for *Isl1* (*ISE*) which is required for normal SAN development and function.

A combinatorial approach incorporating ATAC-seq, chromatin immunoprecipitation, whole-genome bisulfite sequencing, and chromosome conformation capture (Hi-C) was employed by Yang and colleagues to generate a comprehensive map of *cis*-regulatory elements among eleven tissues from adult zebrafish (brain, testis, skin, muscle, heart, kidney, spleen, blood, liver, intestine, and colon) and two embryonic tissues (brain and muscle) [74]. Firstly, *cis*-regulatory elements were defined with the following combinations of histone modifications and ATAC-seq peaks: 25,593 active promoters (H3K27ac, H3K4me3, and ATAC-seq), 40,220 weak promoters (H3K4me3 and ATAC-seq), 58,065 active enhancers (distal H3K27ac and ATAC-seq), and 112,445 heterochromatin (H3K9me2 or H3K9me3) sites. Evolutionary conservation with human and mouse were used as criteria for narrowing down candidates of regulatory sequences. Validation of the predicted enhancers in zebrafish embryo reporter assay identified five putative cardiac enhancers associated with loci encoding well known cardiac TFs Hand2, Gata5–6, Tnnt2a, and Myh6, drove exclusively cardiac expression, while two other were co-expressed together in spleen and muscle. Thus, the comprehensive annotation of the zebrafish genome, gene-regulatory networks, and 3D genome structures underlined evolutionarily conserved elements of genome organization between zebrafish and mammals. The breadth and depth of the data generated by such analysis will help to establish human disease models based on the genomic elements and their structures.

An investigation of deeply conserved enhancers regulating early stages of heart development in the zebrafish was performed by Yuan and colleagues [101]. A transgenic zebrafish line expressing EGFP driven by the smarcd3-F6 enhancer, which labels cardiac progenitor cells prior to *Nkx2.5* expression in mouse embryos [137], was used as an in vivo marker. EGFP signal could be detected as early as 6 h post-fertilization (hpf) along the embryonic margin, which contains mesendodermal progenitors including future cardiac cells. By early somite stage (13 hpf), the expression encompasses almost all cardiac mesoderm expressing *nkx2.5*. Through combined bulk mRNA-seq, ATAC-seq at 10 hpf, single-cell mRNA-seq on 96 hpf. and motif analyses, they uncovered more than 6000 zebrafish open chromatin regions that overlapped with that of human or mouse, representing putative enhancers. Of these, 162 were unique to cardiac progenitor-cell enriched population. Eighteen out of the 21 human-zebrafish conserved regions (accessible CNEs, aCNEs) tested in zebrafish in vivo reporter assay drove heart expression, of which 11 were located near known cardiac genes, including nine which overlapped the experimentally determined binding sites of one or more cardiac TFs (GATA4, NKX2.5, TBX5, HAND2, MEF2A, and SRF) in mouse hearts or cardiac cell types. Both human and zebrafish sequences of three aCNEs found near the essential cardiac genes *hand2/HAND2*, *tbx20/TBX20*, and *mef2cb/MEF2C* were able to drive robust and specific heart expression in stable transgenic lines, demonstrating evolutionary conservation of their function in early cardiac development. Transcriptional repressors (CTBP2, SIN3A, REST, and KAP1) and dual regulators (TCF7L2 and YY1) were found to occupy the aCNEs. Moreover, the binding motifs of many TFs (GATA2, FOXP2, and NANOG) and regulators of chromatin architecture (RAD21 and CTCF) were significantly enriched among the aCNEs, suggesting that they may play diverse roles in gene regulation. Overall, the study supports the existence of CNEs that are primed early in development. Several of them are established in the early embryo and drove tissue-specific gene expression patterns before, during, and later in development. The genomic and epigenomic repertoire of aCNEs suggested that many of them may serve as lineage-restricted enhancers that facilitate the expression of cardiac developmental genes.

### 2.6. Enhancers Direct Gene Expression in the Regenerating Zebrafish Heart

**Figure 2:**
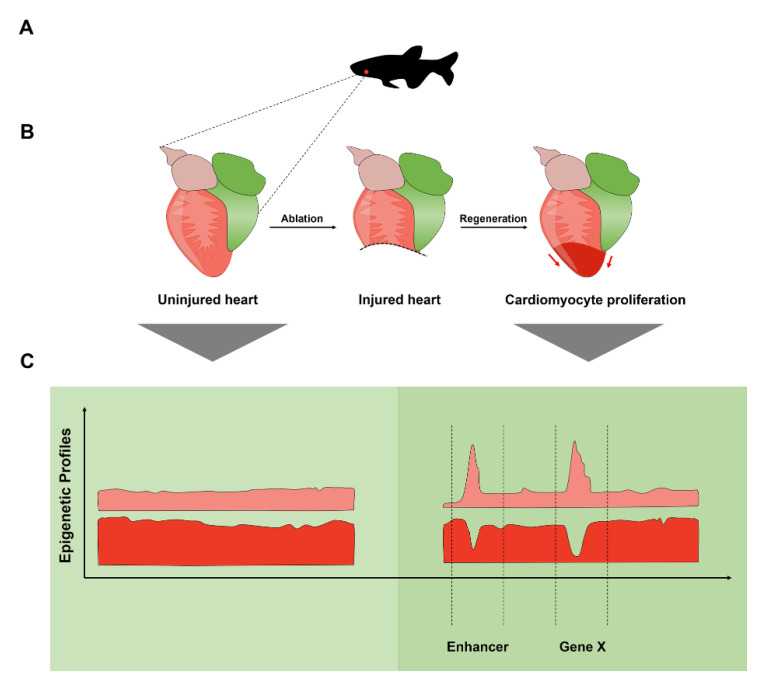
Zebrafish gene regulatory programs of heart regeneration. (**A**,**B**) Zebrafish mature heart: uninjured, injured heart after ablation, and regenerative heart by cardiomyocyte proliferation. (**C**) Enhancers that have no detectable activity in uninjured heart direct gene expression during heart regeneration.

Regeneration is the replacement process of lost or damaged tissue. Many organisms have the capacity of tissue regeneration, including non-mammalian vertebrates such as amphibians and teleost fish which possess the regenerative potential to regenerate body portions, whole limbs, part of heart, and transected spinal cord. Several organ systems, including the brain, spinal cord, heart, and joints, possess minimal regenerative capacity [138]. Many studies have demonstrated that enhancers orchestrate gene expression during tissue regeneration [138,139,140,141]. The adult zebrafish possess a remarkable capacity to regenerate damaged hearts, which makes this organism a powerful model system for deciphering the mechanisms underlying heart regeneration [141] (Figure 2). This process is characterized by a reactivation of certain embryonic gene expression programs. In fact, zebrafish CMs after cardiac injury start to express cardiac TFs known for their role in embryonic heart development such as Nkx2.5 and Tbx20 and Gata4 [142].

Through mapping of dynamic histone modifications, Kang and colleagues [139] identified enhancers that direct gene expression in the regenerating heart and fins, following injury and damage to tissues. They identified 2408 and 859 genes with significantly higher expression in tail fins four days post-amputation and cardiac ventricles seven days after induced genetic ablation of half of all cardiomyocytes. Under normal conditions, *leptin b* (*lepb*) is not highly expressed. However, its expression was induced in the regenerating fins as well as endocardium and in the endothelial lining of inner myofibers. To functionally characterize the regulatory region of *lepb*, the authors generated a zebrafish transgenic line in which the first exon of *lepb* was replaced with an *EGFP* reporter transgene containing 105 kb of DNA sequence upstream of the *lepb* start codon. The transgenic *lepb:eGFP* larvae had little or no detectable EGFP throughout life. Two regions located 7 and 3 kb upstream of the *lepb* start codon displayed enrichment of H3K27ac marks in regenerating, but not in uninjured samples. To map the enhancer responsible for the regeneration specific enhancer activity, the authors established several transgenic lines containing 2, 6, and 7 kb upstream sequences of *lepb* fused to an *EGFP* reporter gene, which identified a short DNA element located 7 kb upstream of *lepb* coding sequence. This element, referred to as *lepb*-linked enhancer (LEN), could direct regeneration-activated gene expression from multiple promoters. In addition, they provided evidence on other LEN fragments located more proximally (comprising approximate nucleotides 830–1350 or 1000–1350) which directed endocardial expression during heart regeneration. Their results show that distinct elements regulate heart and fin gene expression program during regeneration. In addition, the heart and fin enhancers could activate gene expression in injured neonatal mouse tissues, showing that they are functional in a species that does not have strong regenerative capabilities in these organs. This observation demonstrates that the LENs are conserved in function within the context of tissue regeneration, which further led to the question of whether they are also present in mammals or may have been lost during evolution, resulting in a reduced regenerative capability in these organisms.

Further analysis of the *lepb* regeneration enhancer was performed by Begeman and colleagues [143], who molecularly dissected the LENs further to decipher how they instruct regeneration-dependent gene expression in the heart. They focused on a 317 bp cardiac LEN (cLEN) enhancer fragment, which was used to drive EGFP reporter in the transgenic reporter zebrafish line cLEN:EGFP which carried cLEN coupled to the *lepb* 2 kb minimal promoter (P2) and *EGFP* cassette. EGFP was undetectable in uninjured hearts but was strongly induced upon ablation, indicating that cLEN is not a developmental enhancer in the heart and that injury signals are required for its activity. cLEN was found to contain binding sites for Nfat, Gata, Fox, and Ets TFs which were associated with endothelial/endocardial cells. To establish the minimal core for enhancer activity, various fragments of the cLEN was used to drive EGFP reporter in zebrafish transgenic lines, which revealed that at least two regulatory elements within the cLEN region are required for regeneration-dependent activation. In addition, they discovered that a 22 bp sequence in cLEN suppresses regeneration enhancer activity, providing evidence that cardiac regeneration enhancers are actively repressed in uninjured and regenerating hearts to prevent aberrant gene expression. These results show that modulation of gene expression and regenerative potential in injury sites may be triggered by tissue regeneration enhancer elements.

Along the same path of the tissue repair enhancer elements, Goldman and colleagues [144] generated a high-resolution resource of gene regulatory changes in CMs during the process of heart regeneration. First, they used the promoter of *cardiac myosin light chain2 (cmlc2)* to drive cardiomyocyte-restricted expression of histone H3.3 fused with biotin ligase BirA in a transgenic zebrafish line *cmlc2:H3.3-bio*. This enabled them to isolate H3.3-biotin-enriched open chromatin regions from ventricles. H3.3-bio was enriched in regions containing genes expressed in cardiac cell types, such as the TFs *gata4*, *hand2*, and *meis1b*. In total, 35,127 H3.3 peaks overlapped with regions marked by H3K27Ac, which indicates active enhancers. Regions with only H3.3 marks contained binding sites for Gata4, Meis1, and Mef2d. Enhancer activity assay of selected candidates confirmed that the enrichment of H3.3 alone was a reliable marker of enhancer activity. A global increase of H3.3 enrichment was observed in regenerating hearts, indicating that changes in CM chromatin structure occur during regeneration. Interestingly, 84% of genes which contained H3.3 at their promoters in the uninjured profile have decreasing levels of H3.3 during regeneration. Differential H3.3 peaks were found in and around many genes known to be induced during heart regeneration, including *gata4*, *tbx5a*, *tbx20*, *stat3*, *nppa*, and *nfkbiab*. To identify enhancer elements that could be responsible for changes in CM gene expression during regeneration, they examined intergenic DNA regions that increased in H3.3 occupancy during regeneration. They found 11,964 intergenic H3.3 peaks (28% of total) arising de novo in the vicinity of 5232 genes. Of these genes, many also changed in expression and had increasing enrichment of H3.3 at their promoters or in gene bodies, suggesting possible cis regulation by these nearby intergenic sequences. To validate the activity of these H3.3 peaks as regeneration enhancers, they tested 28 of these elements in a zebrafish transgenic reporter assay and created stable transgenic lines. Among these transgenic lines, 23 expressed EGFP in various tissues of larval zebrafish, three of which had cardiac expression. Three enhancers were located 103 kb upstream of the *runx1* gene, ~50 kb upstream of *kcna1* and/or *kcna6a* gene, and ~5 kb upstream of anillin drove minimal or sporadic EGFP signal in uninjured heart, while ablation injury induced EGFP throughout regenerating CMs. Another enhancer located 22 kb upstream of the *sema3aa* gene expressed EGFP throughout the ventricle only after genetic ablation injury. Thus, the authors showed that H3.3 profiling can be used to find previously unknown *cis*-regulatory related to heart regeneration. The discovery of enhancers driving cardiac regeneration contributes valuable insights into the mechanism of cardiac tissue repair which has important implications in treatment of cardiac injury in humans.

## 3. Technological Advances in Enhancer Discovery Provides Future Opportunities for Identification of Cardiac Enhancers

As discussed in the preceding sections, cardiac enhancers study in model organisms has benefitted from recent methodological advances which allowed for their rapid discovery and functional testing. In this section, we review promising techniques and approaches which have been utilized for enhancer studies in other systems and could potentially be applied to cardiac biology. Many of these techniques pose the issue of requiring large amounts of input materials (such as the detection of eRNAs by CAGE) which is challenging for cardiac samples originating from primary tissues, or the necessity of relevant cardiac datasets from model organisms, which is still scarce. Nevertheless, as more datasets on cardiac enhancers from in vivo studies will emerge, it is expected that new techniques, particularly computational modeling, would become increasingly relevant. The zebrafish provides opportunity for the application of such large-scale analysis in studying early heart development given its ability to produce large numbers of embryos and its external development. Moreover, the initiation of the DANIO-code consortium effort to explore its genome in greater detail promises to make more genomic datasets available for this useful model organism.

### 3.1. Transcription of Enhancer Regions Pinpoints the Presence of Active Enhancers

**Figure 3:**
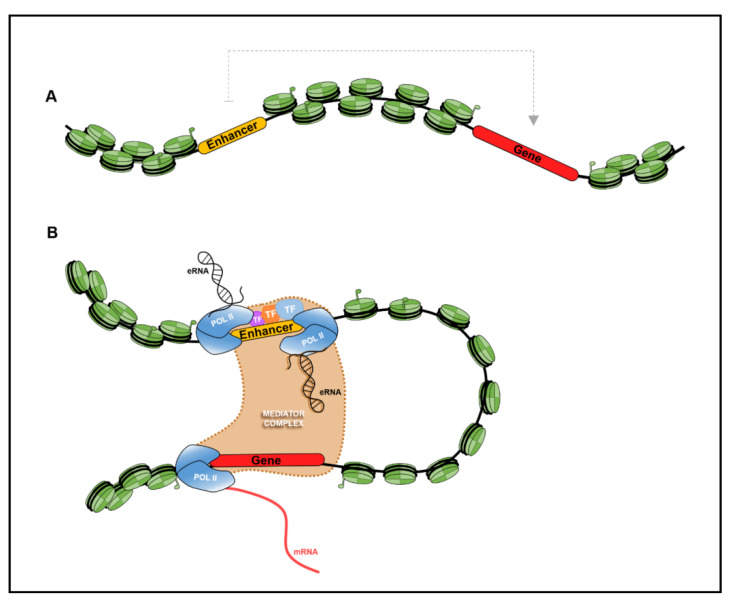
Model of enhancer RNA mechanism. (**A**) Enhancer and gene sequences in chromatin prior to gene activity. (**B**) Partially or fully assembled sets of TFs can associate with the enhancer and the gene promoter. In the active state, the enhancer and its transcript may physically associate with the gene, triggering formation of a mediator complex [20,21,22,23,24].

One of the defining characteristics of enhancers that emerged in the recent years is their bidirectional expression, producing unspliced, polyadenylated, and relatively unstable transcripts known as enhancer RNAs (eRNAs) (Figure 3). This phenomenon was first reported by Francesca De Santa and colleagues in 2010 [21], revealing widespread transcription of extragenic genomic sequences which did not overlap with protein-coding genes in LPS-interferon-gamma (*IFNγ*) activated primary mouse macrophages. RNA-Pol II recruitment sites were observed on distal, non-genic, locations that are bound by epigenetic marks (mainly, H3K4me1, H3K27ac, and P300, recognized as hallmarks of active enhancers) in unstimulated and stimulated macrophages. In total, 4588 extragenic Pol II peaks upstream of various immune-related genes were detected upon stimulation of immune cells. A machine learning approach relying on specific chromatin signatures was employed to classify these peaks into enhancer or promoter elements, which identified 3227 peaks originating from enhancer-specific regions and 1004 peaks associated with promoters. This study provided insights into possible regulatory mechanism of enhancers by means of its transcription and reflects the pervasive transcription within the genome of complex systems. Genome-wide enhancer transcription was subsequently corroborated in mouse cortical neuronal cells upon stimulating membrane depolarization [136]. The authors employed ChIP-seq to profile open and active chromatin state, and RNA Pol II occupancy. Transcripts originating from the enhancer regions were detected by obtaining the genome-wide distribution of CBP, a transcriptional co-activator which mediates the binding of RNA Pol II at the site of eRNA transcription, and RNA Pol II binding sites within enhancer elements. Strikingly, ~2000 out of ~5000 discovered extragenic enhancer regions were found to produce short eRNAs. Similarly, ~1000 out of ~7000 intragenic enhancer regions were transcribed into short eRNAs. Robust correlation was observed between the levels of eRNA transcription and expression of genes located in their proximity. Moreover, the binding sites of various neuron-related TFs such as cAMP-response element binding protein (CREB) and Neuronal PAS Domain Protein 4 (NPAS4) were found within 100 bp of the enhancer regions. Importantly, their analysis also required enhancer–promoter loop formation to transcribe enhancer DNA sequences. Together, their results demonstrate the in vivo transcription of enhancer sequences, yielding RNA Pol II- and promoter-dependent non-polyadenylated, relatively unstable yet functional eRNA, that participate in regulatory processes of the neuronal systems. Subsequent studies have attempted to elucidate the mechanism and function of these bidirectional enhancers and readers are directed to excellent reviews by [145,146,147,148].

Taking advantage of bidirectional transcription feature, enhancers can therefore be detected using methods that precisely detect non-promoter TSSs at exceedingly high resolution. One example of such method is cap analysis of gene expression (CAGE) which relies on capturing the 5′-end of capped, polyadenylated and non-polyadenylated transcript species, screening the entire landscape of cell transcriptome [149,150]. Using this powerful approach, the discovery of known as well as novel TSS, including those of enhancer regions, can be achieved [151,152,153]. To this end, Andersson and colleagues [94] published an inventory of 43,011 active enhancers based on bidirectional transcription marks. They carried out an integrative analysis of CAGE-seq data (obtained from Functional ANnoTation Of the Mammalian genome 5 (FANTOM5) Consortium project [154,155,156]) across 432 human cell-types, 135 different tissues from 241 cell line samples to annotate cell-type specific enhancers. Altogether, this approach predicted 43,011 enhancers. Another study utilized the bidirectional transcription phenomenon to examine the regulatory significance of eRNA formation during osteoclasts differentiation process by analyzing CAGE libraries from mice in-vitro differentiated osteoclasts of bone-marrow-derived monocyte-macrophage precursor cells (BMMs) [95]. CAGE data analysis identified 132,744 TSSs, of which 19,171 eRNA candidates were identified with bidirectional transcription pattern within a maximum of 300 bp. Interestingly, the bidirectional transcripts of 87 of these loci were more than 10-fold increased upon stimulation of the precursor cells, implying their role osteoclastogenesis.

The short life span of eRNAs poses a challenge in capturing the entire landscape of this class of transcripts. Sequencing pre-mature or nascent transcripts, which are either in the phase of on-going transcription or just transcribed and have not undergone the splicing process, has been shown to circumvent this problem. Mayer and colleagues [157] established a method called native elongating transcript sequencing (NET-Seq) to segregate nascent transcripts followed by sequencing of their 3′-ends to gain insights about RNA Pol II progression at genome-wide scale. Through NET-seq application, high-resolution map of RNA Pol II occupancy could be obtained. More recently, Hirabayashi and colleagues [8] established a modified version of this method by combining it with CAGE, known as NET-CAGE where they sequenced 5′-ends of the nascent transcripts to precisely detect enhancer transcribed RNAs. A combination of NET-CAGE and CAGE analysis in MCF-7 breast cancer cells found 7305 FANTOM5 enhancers. Notably, 3977 enhancers were found from NET-CAGE alone, while CAGE discovered only 272 FANTOM5 enhancers. On the contrary, Young and colleagues [158] questioned the reliability of enhancer identification by analyzing bidirectional transcription signals alone. From the multi-omics data analysis, displaying chromatin accessibility patterns, DHS and CAGE transcription initiation loci within Gm12878, HepG2, Huvec, and K562 cell-lines, the authors pointed out the co-existence of bidirectional transcription signals around DHSs midpoints with or without chromatin accessibility marks which indicates that such signals do not explicitly mark the enhancer regions. Furthermore, their study also implies the irrelevance of pervasive transcription to some degree towards the regulation of cellular gene expression.

### 3.2. Computational Modeling Approaches Allows Integrative Analyses of Genomic Data for Large Scale Enhancers Discovery

Computational methodologies have been developed to distinguish between different genomic features within accessible chromatin regions (Table 2). These include TF footprinting algorithms, which identify TF binding based on the detection of cleavage-protected “footprints” within the area of accessible chromatin which result from the binding of single or clusters of TFs. As a result, among the otherwise high read coverage regions overlapping accessible regions, footprints show abrupt decrease in read coverage due to presence of certain TFs. Several DNaseI-seq- [159,160,161,162] and ATAC-seq-based [163,164,165,166] computational footprinting methods aid the identification of TFBS orchestrated within the regulatory regions, suggesting the presence of enhancers.

Machine-learning-based computational modeling has been increasingly applied to effectively discover functionally relevant regulatory regions from high-throughput genomic datasets generated by large-scale efforts such as ENCODE [75,121,122,123], NIH Roadmap project [126], and FANTOM [151,154,155,156]. Various methods have been developed based on Gibbs sampling and linear regression classifiers, hidden Markov models, artificial neural network-based models, deep-learning algorithms, deep neural network, support vector machine framework (and its flexible variant called multiple kernel learning, MKL), dynamic Bayesian network (DBN), and random forest method to predict enhancers and other functionally significant non-coding elements with high sensitivity and specificity. Supervised machine learning methods rely on a training set from experimentally validated sequence characteristics, and these signatures are further used to predict uncharacterized (test) genomic features of interest. In enhancer prediction, the training set includes all the enhancer-related (positive training set) and non-enhancer-related (negative training set) signatures to predict the enhancers and non-enhancers in the test set. Various genome-wide signatures are employed by machine learning computational models to predict enhancer sequences, including enhancer-specific histone modification profiles (H3K27ac and H3K4me1), P300 transcription co-activator binding site enrichment, DHSs, TF binding motifs along with specific interactions with their TFs, active chromatin accessibility regions, and CAGE tags covering bidirectional transcription demonstrating bimodal distribution. Some methods rely on single identification signatures such as histone modifications, phylogenetic sequence conservation, or dense cluster of TF binding motifs within *cis*-regulatory regions, while others count on multiple feature detection, making them more robust.

A supervised computational approach to discover functional regulatory elements was introduced by Heintzman and colleagues [91] based on two histone modification profiles obtained by interrogating a 30 Mb (~1%) sized human genome. The method was used to predict profile-based enhancer and promoter sequences regulating gene expression within human HeLa cells. However, profile-based prediction methods rely on a limited few chromatin histone modification. In addition, a smaller window size of 10 kb for searching the peaks impacts the overall detection signal. To improve the enhancer prediction approach, Won and colleagues [167] designed another supervised learning method based on hidden Markov model integrated with simulated annealing approach (HMM-SA) to effectively locate enhancer and promoter regions within ENCODE data. HMM-SA discriminates between enhancers and promoters based on combinations of histone marks denoting functional regulatory elements. They utilized 73 enhancer and 107 promoter profiles to train their model. Both the profile-based model discussed above and HMM-based methods resulted in similar numbers of putative enhancers (~82% common enhancers in ENCODE regions). However, HMM method predicted a higher number of TSS compared to profile-based approach, and its predictions were supported by other marks such as CAGE tags, P300, and DHSs. Furthermore, several popular HMM and their generalized form, dynamic Bayesian networks (DBN)-based mathematical models, have been developed for genome-wide enhancer prediction using either supervised (such as CHROMatin-based Integrated Approach (Chromia) [168] and enhancer-HMM [169]) or unsupervised (such as ChromHMM [170], Genostan [171], and Segway [172]) learning algorithms. The ENCODE project consortium [173] implemented an unsupervised machine learning method to annotate functionally relevant regions across 1640 genomics datasets in 147 distinct human cell-types, generated under this project. For training their models, they used ChromHMM [170] and Segway [172] to obtain comprehensive functional annotations in human genome. Around 13,000 putative enhancers were discovered using this approach.

Artificial Neural Networks (ANN) are also being utilized for data classification means. Because of this, Firpi and colleagues [174] developed a computational framework based on chromatin histone modification marks to confidently characterize genomic regulatory elements (mainly enhancers). The method is called CSI-ANN (chromatin signature identification by artificial neural network). Energy and mean functions were used to transform the raw data (containing chromatin 39 histone modification marks, P300 binding sites) into feature values to enhance the signal-to-noise ratio in enhancer identification. Further, to effectively reduce the dimensions (feature space reduction) of calculated feature values from the given histone modifications data, Fisher discriminant analysis (FDA) was performed. Then, time-delay neural network (TDNN) was used as classification technique, which incorporates epigenetic states as input. CSI-ANN was applied on human CD4+ cells where the positive training set contained 213 enhancers while 2130 sequences comprised the negative training set. In total, 36,769 T-cell specific enhancers were predicted in this experiment.

Rajagopal and colleagues [175] presented a Random-forest-based enhancer prediction model by using chromatin states data, along with distal P300 binding sites profiles, for enhancer prediction across 12 distinct cell-types from ENCODE database. To classify enhancer from non-enhancer features, the algorithm builds multiple random decision trees, one for each sample, which works in an ensemble to perform predictions at each decision tree and then vote for the best mostly observed outcome. Fernandez and colleagues [176], devised an approach for chromatin state detection using SVM combined with genetic algorithm optimization (ChromaGenSVM). Using a set of 38 histone modification signatures in human CD4+ T cells, the algorithm is trained to identify chromatin marks classify enhancers with high confidence. Five histone marks (H3K4Me1, H3K4Me3, H3R2Me2, H4K8Ac, and H2BK5Ac) were qualified by the model to successfully discriminate the active enhancers. Using this approach, they predicted 23,574 enhancers, out of which 89% were supported with DHSs experimental data, 31% had P300 binding sites, 11% enhancers were enriched with TF binding motifs, and 10% were found to have evolutionary conservation in 17 vertebrate species.

EnhancerFinder [177] applies a SVM-centered method named multiple kernel learning (MKL) which perform two rounds of supervised machine learning steps to predict tissue-specific enhancers. The model’s training classifier was obtained from VISTA enhancer browser [178]. This method integrates distinct datatypes including sequence conservation, TFB motifs, and functional genomics data (such as ChIP-seq and DNase I-seq). Applying this method at genome-wide scale in humans, Erwin and colleagues identified >80,000 developmental enhancers. The weakness of this approach is its dependency upon VISTA data resource for training classifier. Kleftogiannis and colleagues [179] presented DEEP, another SVM-based framework to predict enhancers. This method comprises three distinct variations based on the data resource being used to train the model classifier: DEEP-ENCODE, DEEP-VISTA, and DEEP-FANTOM5. Additionally, unsupervised Bayesian framework-based methods have also reported successful classification of regulatory regions. A method called, cisTopic [180] relies upon the mathematical modeling of single-cell epigenomics data (scATAC-seq) to detect co-accessible enhancers by taking cellular diversity into account.

Besides the prediction of enhancers, some mathematical models also detect the strength of the predicted enhancers. For example, iEnhancer-2L [181], which has the potential to detect enhancers with an additional yet critical information about their potency (strong or weak enhancer). Its uses pseudo k-tuple nucleotide composition to obtain features for enhancer prediction (73% precision) and classification (60.5% precision). Similar methods include EnhancerPred [182], EnhancerPred2.0 [183], and iEnhancer-EL [184]. Earlier this year, Zhang and colleagues [185] established another method to predict the strength of enhancer sequences by implementing Augmented data and Residual Convolutional Neural Network, abbreviated as ES-ARCNN.

Sequence level disruptions within enhancer regions could lead to common and complex diseases. Hence, it is extremely critical to develop computational methods to rapidly analyze multi-omics data obtained from clinical samples to predict the impact of such disruptive genetic variations, causing malfunctioning of regulatory regions. Various methods have been devised to quantify the impact of single nucleotide variations within functional regulatory elements, including enhancers. Thus, Lee and colleagues [186] developed a computational approach called DeltaSVM, a gkm-SVM-based computational method to detect the consequences of SNPs within the *cis*-regulatory regions localized distally to TSS. They utilized the DNase I–sensitivity quantitative trait loci (dsQTLs) dataset which was generated for human lymphoblastoid cell lines (LCLs). The SVM model was trained on DHSs data for each possible 10-mer sequence and calculates a collective weight to further quantify the impact of SNPs in chromatin accessibility by applying DeltaSVM. Recently, Thibodeau and colleagues [187] developed a neural network-based method to assimilate ATAC-seq data derived from pathological conditions with DNA sequence content to reveal the enhancer variability, associated with diseases. The method is called as Predicting Enhancers from ATAC-Seq data (PEAS).

## 4. Conclusions and Future Perspectives for the Role of Enhancers in Human Health and Diseases

The central idea that genes were the prominent elements driving protein expression was revolutionized by the discovery of other elements that had a regulatory action on them. While genes were considered to be the head of the transcriptional and translational mechanism, regulatory elements such as enhancers can be considered as the neck of this mechanism capable of making the head move in the direction they wanted. With the availability of methods to detect and assess their function, the role of enhancers as regulators of gene expression profiles became increasingly apparent and easier to detect. Comparative studies between phylogenetically close or distant organisms have revealed deeply conserved enhancers essential for development. In addition, it has become increasingly clear that enhancers dynamically transit through active and poised state in development. Thus, it would seem reasonable to think that the development of the heart from cardiac progenitor cells to the contractile mature heart is accompanied by dynamic changes in gene regulatory landscape. Many studies reported here have highlighted the dynamic activity of enhancers, particularly in the hearts of different organisms such as mammals and fish.

Given its role in regulating precise gene expression, the importance of enhancers in development and disease has gained increasing recognition. Many studies investigated the impact of single nucleotide variations within human enhancer elements on their regulatory function and, further, its impact on the enhancer transcription. Such perturbations are found to be implicated in the onset of complex diseases including congenital heart diseases. For instance, mutation in enhancers of the *TBX5* or *NKX2.5* gene could result in human congenital heart phenotype [188,189,190]. A comprehensive knowledge on enhancers implicated in heart development and function would therefore help to expand our capacity to diagnose and develop more precise treatment for heart disorders.

The increasing availability of genomic and epigenomic datasets on various human tissues and model organisms enabled the identification of such candidate enhancers through integrated computational analyses. However, despite the rapid advances of techniques for large-scale detection of putative enhancer regions, assigning function and mechanism to them still faces challenges, mainly due to the fact that enhancers are orientation independent and can be located anywhere up to ~1 Mb [191] from their target gene promoter in vertebrate genomes. Several approaches have been employed to tackle this issue, including computational modeling approaches. In one example, a “scoring method” called Predicting Enhancer Gene Associations Using Synteny (PEGASUS) was developed based on the assumption that the physical link between them and their target genes are also conserved [192]. The algorithm therefore identifies syntenically conserved enhancer–target gene pairs across multiple vertebrate species. Application of this method on whole human and zebrafish genome [193] identified more than one million enhancers linked to almost 20,000 protein coding genes in both species. Other approaches to establish enhancer–target gene link, which are not covered in this review, include the use of chromatin conformation capture methods to capture physical links between enhancer and its target gene promoter [194,195,196].

The ability to link enhancers to their putative targets provide critical insights into their possible role in specific biological processes, particularly if the function of the target is already known. However, validating the enhancer activity of these millions of regions still poses an uphill task. The availability of in vivo enhancer assay in model organisms, particularly the zebrafish, have allowed rapid screening of enhancer candidates, further narrowing down relevant candidates for further analysis, as described in several examples in this review. Stable transgenic animals could subsequently be generated which expresses reporter genes under the control of the identified enhancer, which allows for more detailed functional characterization before going back to the human patients to perform targeted sequencing of the validated regions. In addition, discovery and functional analyses of conserved enhancers in model organism through experiments on native embryonic heart tissues would allow us to fill the remaining knowledge gap on enhancer function in vivo, during the active developmental period when gene regulation is presumed to be most dynamic.

Given its role in regulating precise gene expression, the importance of enhancers in development and disease has gained increasing recognition. Many studies investigated the impact of single nucleotide variations within human enhancer elements on their regulatory function and, further, its impact on the enhancer transcription. Such perturbations are found to be implicated in the onset of complex diseases including congenital heart diseases. For instance, Smemo and colleagues [188] searched for regulatory mutations impacting the activity of TBX5. Using a combination of genomics, bioinformatics, and mouse/zebrafish genetic engineering, ~700 kb of the *TBX5* locus was scanned to search *cis*-regulatory elements. They highlighted that a significant number of CHD associated with TBX5 dysfunction might arise from non-coding mutations in TBX5 heart enhancers. In addition, mutation in an upstream enhancer of the human *NKX2.5* gene resulted in ventricular septal defects [188,189,190]. These examples therefore illustrate that a comprehensive knowledge on enhancers implicated in heart development and function would significantly expand our capacity to diagnose and develop more precise treatment for heart disorders.

In summary, an integrative approach from computational to clinic has advanced our understanding of enhancers role in development and disease. The remaining task at hand is to elucidate the mechanism by which they contribute to development and disease. CRISPR-based DNA editing has significantly expanded our toolkit for functional characterization of various coding and non-coding DNA elements. Recently, various applications of CRISPR/Cas9 have been developed for large-scale screening of enhancer function genome-wide [197,198]. This approach allows direct functional assay within the native endogenous conditions which is lost in traditional reporter assays. Further applications of CRISPR-based large-scale screening methods therefore promises to advance the discovery of functional enhancers and their mechanism of action.

## Figures and Tables

**Table 1:** A list of methods known for enhancer identification.

Biological Approaches	Work-Principle	Reference
Enhancer-Deletion Approach	Deletion of non-coding *cis*-regulatory DNA elements can severely disrupt the systemic functions.	[83]
Enhancer-Trap Assay	Through microinjection of embryos random integration of a vector-construct with minimal promoter and reporter gene, driving expression if enhancer is present.	[84,85,86,87,88]
Transient Transfection Assay	Luciferase reporter plasmid constructs containing promoter and 5′-flanking DNA sequence increases the luciferase expression in the presence of enhancer region.	[81]
**High-Throughput Techniques**		
DNase I-seq	DNase I digestion and DHS fragments mostly comprises *cis*-regulatory regions (e.g., enhancers).	[89]
Epigenomic Profiling (ChIP-Seq)	Enrichment of H3K4me1, H3K4ac, and P300 histone modifications determines the active enhancers.	[20,55,56,60,90,91,92,93]
CAGE	High resolution map of TSS and bidirectional transcription patterns defines the precise location of enhancers.	[94,95]
NET-CAGE	Capturing 5′-ends of nascent transcripts by fusing two technologies helps to identify unstable transcripts (eRNA).	[8]
ATAC-Seq	Accessible chromatin regions encompass enhancer elements.	[58,96]

**Table 2:** In-Silico Algorithms for Enhancer Prediction.

Mathematical Model	Algorithm	Reference	Link
**Supervised Machine Learning (Probabilistic Graphical Models)**			
HMM-SA	An HMM-based classifier obtained for enhancer, promoter, and background. Individual log-odd score measurement for each classifier for a genomic region of interest and score is averaged over three quantified scores. Simulated annealing algorithm implementation to obtain best combination of histone modification marks defining enhancers.	[167]	http://http:/nash.ucsd.edu/chromatin.tar.gz (accessed on 9 April 2021)
CHROMatin based Integrated Approach (Chromia)	Parallel HMM model combines histone modifications data and genomic sequence (motif information) to perform predictions. Computation of position specific scoring matrices (PSSM Scores).	[168]	http://wanglab.ucsd.edu/star/ (accessed on 9 April 2021)
enhancer-HMM	A probabilistic model based on HMM. Training is performed with histone modification marks data (ChIP-Seq) and chromatin accessibility data (ATAC-Seq).	[169]	https://github.com/tobiaszehnder/ehmm (accessed on 9 April 2021)
**Unsupervised Machine Learning**			
ChromHMM	Application of multivariate HMM to train the classification model. Training is performed on histone modification marks data.	[170]	http://compbio.mit.edu/ChromHMM/ (accessed on 9 April 2021)
GenoStan	Genome segmentation-based method with HMM application. Read counts modeled with Poisson lognormal and negative binomial distribution approaches. Model’s parameter training solely relies upon the given raw data without automation on chromatin states (manual parameter). Model training using ChIP-Seq and DNase I-Seq chromatin marks.	[171]	http://bioconductor.org/packages/3.4/bioc/html/STAN.html http://i12g-gagneurweb.in.tum.de/public/paper/GenoSTAN (accessed on 9 April 2021)
Segway	An application of unsupervised genome segmentation approach using dynamic Bayesian network algorithm. Integration of ChIP-seq, DNase I-Seq, transcription factor and FAIRE-Seq data. Model training on 1% of human genome with ChIP-Seq, Dnase I-Seq and FAIRE-Seq data from ENCODE pilot project. Viterbi decoding helped to identify genome segments of 2 Mb size.	[172]	https://pmgenomics.ca/hoffmanlab/proj/segway/ (accessed on 9 April 2021)
cisTopic	Model training on single-cell ATAC-Seq data using unsupervised Bayesian framework. Probabilistic modeling with latent Dirichlet allocation with a collapsed Gibbs sampler.	[180]	http://github.com/aertslab/cistopic (accessed on 9 April 2021)
**Artificial Neural Networks (ANN)**			
Chromatin Signature Identification by Artificial Neural Network (CSI-ANN)	Time-delay neural network was applied for feature classification task.Mathematical functions: Mean and Energy are utilized to transform genome-wide data. Fisher discriminant analysis is performed to convert the high dimensionality of data to enhance the accuracy of classification model. Model is trained on histone modifications data.	[174]	http://www.medicine.uiowa.edu/Labs/tan/CSIANNsoft.zip (accessed on 9 April 2021)
Random Forest based Enhancer identification from Chromatin States (RFECS)	Random forest-based mathematical model is utilized to classify features. Model is trained on ENCODE chromatin modifications data and DNase I-Seq data.	[175]	http://enhancer.ucsd.edu/renlab/RFECS_enhancer_prediction/Training (accessed on 9 April 2021)
**Support Vector Machine**			
ChromaGenSVM	Chromatin state detection using support vector machines in combination with genetic algorithm optimization. Model is trained with ChIP-chip data from ENCODE project and ChIP-Seq data containing DNA-methylation and acetylation marks.	[176]	http://sysimm.ifrec.osaka-u.ac.jp/download/Diego/ (accessed on 9 April 2021)
EnhancerFinder (multiple kernel learning)	It works by incorporating multiple datatypes in the prediction process such as chromatin modification marks, sequence-level conservation, and DNA sequence motifs. Model is trained by using developmental enhancers from VISTA enhancer browser.	[177]	Putative enhancer elements are available at UCSC genome browser (accessed on 9 April 2021)
DEEP	It comprises three main components: DEEP-ENCODE, DEEP-FANTOM5 and DEEP-VISTA. Application of both SVM and ANN to train the prediction model.	[179]	http://cbrc.kaust.edu.sa/deep/ (accessed on 9 April 2021)
TF Footprinting	Nucleosome bound DNA restricts its cleavage, producing low signal. Similarly, open chromatin regions (with high signal) are bound by TFs tend to restrict cleavage, generating weak signal. These regions are referred to as “footprints”, representing the presence of enhancer elements occupied by TFs.	[159,160,161,162,163,164,165,166]	
Sequence-based Evolutionary Conservation	Developmental enhancers are known to be conserved among cross-species genomic sequences.	[103,104,105,106,107,108,109]	
**Enhancers Strength Prediction**			
iEnhancer-2L	A SVM-based model trained on histone modification data. Use of pseudo k-tuple nucleotide sequence composition.	[181]	http://bioinformatics.hitsz.edu.cn/iEnhancer-2L/ (accessed on 9 April 2021)
EnhancerPred	Model is trained on chromatin states. Implementation of Bi-profile Bayes to obtain nucleotide sequence features. Rank the predictions based on F-score.	[182]	http://server.malab.cn/EnhancerPRED/ (accessed on 9 April 2021)
EnhancerPred2.0	A SVM based classification model trained on chromatin modifications data. Integration of position-specific trinucleotide propensity and electron ion-interaction pseudopotential of DNA sequence. Computation of F-score to rank predictions.	[183]	
iEnhancer-EL	A SVM based model. DNA sequence composition and nucleotide frequencies are obtained using Kmer, subsequence and pseudo k-tuple methods.	[184]	http://bioinformatics.hitsz.edu.cn/iEnhancer-EL/ (accessed on 9 April 2021)
enhancer sequences by implementing Augmented data and Residual Convolutional Neural Network (ES-ARCNN)	Implementation of residual convolution neural network to train the classification model. Enlarging the input data using reverse complement and shifting method to gain better predictions.	[185]	http://compgenomics.utsa.edu/ES-ARCNN/ (accessed on 9 April 2021)
**Sequence-level Variation within Enhancers**			
DeltaSVM	Prediction of the impact of sequence variation in enhancer activity. Model is trained with DNase I-seq and ChIP-seq data.	[186]	http://www.beerlab.org/deltasvm (accessed on 9 April 2021)
Predicting Enhancers from ATAC-Seq data (PEAS)	Implementation of neural networks model. Integration of chromatin accessibility data with nucleotide sequence composition (e.g., GC%).	[187]	https://github.com/UcarLab/PEAS (accessed on 9 April 2021)
